# Dosage-Associated Maternal Dominance and Tissue-Specific Paternal Expression in a Triploid Hybrid Between Grass Carp and Yellowcheek

**DOI:** 10.3390/ani16152299

**Published:** 2026-07-24

**Authors:** Shuai Chang, Xiao-Li Yang, Li Zhou, Zhi Li, Peng Yu, Meng Lu, Xi-Yin Li, Zhong-Wei Wang, Xiao-Juan Zhang, Jian-Fang Gui, Yang Wang

**Affiliations:** 1 College of Fisheries and Life Science, Dalian Ocean University, Dalian 116023, China; 2 State Key Laboratory of Breeding Biotechnology and Sustainable Aquaculture, Hubei Hongshan Laboratory, The Innovation Academy of Seed Design, Institute of Hydrobiology, Chinese Academy of Sciences, 7 Donghu South Road, Wuhan 430072, China; 3 Hubei Hongshan Laboratory, Huazhong Agricultural University, Wuhan 430070, China; 4 University of Chinese Academy of Sciences, Beijing 100049, China

**Keywords:** cyprinids, allotriploid, allele-specific expression, expression-level dominance, whole-genome resequencing, intestinal histology, goblet cells

## Abstract

Allopolyploidy can generate rapid phenotypic change, yet its early transcriptomic consequences in a newly generated triploid hybrid remain poorly understood in animals. Here, we establish a sterile triploid hybrid between grass carp (*Ctenopharyngodon idella*) and yellowcheek (*Elopichthys bambusa*), designated CCE, using an optimized hydrostatic pressure protocol that achieved 100% triploidy with high embryonic viability. Whole-genome resequencing supported an approximately 2:1 maternal-to-paternal genomic dosage. Transcriptomes from seven tissues revealed that maternal expression-level dominance (ELD-*Ci*) was the predominant expression-inheritance pattern (63.7–84.2%), consistent with a passive, dosage-dependent mode of regulation. Paternal expression-level dominance (ELD-*Eb*) was detected in all tissues but with marked tissue specificity: most ELD-*Eb* genes in the liver and muscle showed expression levels similar to those of the paternal yellowcheek parent before hybridization (96.2% and 72.8%, respectively), whereas the brain and hypothalamus contained more ELD-*Eb* genes not directly explained by parental differences. Finally, in the gut, CCE had shorter villi than both parents, a thicker muscularis layer than both parents, and fewer goblet cells than *Eb*. Genes related to digestion, absorption, and nutrient transport were more highly expressed in *Eb* and retained an *Eb*-like expression pattern in CCE. By contrast, several smooth muscle-related genes, including *myh14* and *smtnl1*, showed non-additive upregulation beyond the parental range, in parallel with the thickened muscularis layer. Together, these findings provide new insights into how parental genome dosage and tissue-specific regulation shape gene expression divergence in newly formed animal allopolyploids.

## Simple Summary

Polyploid fish have been widely applied in aquaculture because certain polyploid lineages exhibit desirable traits, such as sterility and enhanced production performance. In this study, we produced a sterile triploid hybrid between grass carp and yellowcheek, named CCE, using hydrostatic pressure treatment, in which fertilized eggs are exposed to high pressure to induce triploidy. We confirmed that CCE contains two maternal chromosome sets from grass carp and one paternal chromosome set from yellowcheek. To understand how the two parental genomes contribute to gene expression in this new triploid hybrid, we analyzed transcriptomes from seven tissues, including brain, hypothalamus, liver, gut, kidney, spleen, and muscle. The results showed that overall gene expression in CCE was mainly similar to that of the maternal grass carp, which is consistent with its higher genomic dosage. However, paternal yellowcheek also contributed to gene expression in a tissue-specific manner. In particular, the gut of CCE showed shorter villi, a thicker muscularis layer, and altered expression of digestion-, nutrient transport-, muscle-, and epithelium-related genes. These changes suggest tissue-level divergence after hybridization and triploidization, although their functions remain unclear. Overall, this study provides a multi-tissue reference for parental genome interactions in newly formed triploid fish and sterile polyploid fish development.

## 1. Introduction

Allopolyploidy, in which hybridization is combined with genome doubling, brings together divergent parental genomes within a single nucleus and can trigger rapid transcriptomic and phenotypic changes that are not predictable from either parent alone [1,2]. The combined effects of heterosis and polyploidy often confer enhanced agronomic or aquacultural traits [3], as exemplified by domesticated allopolyploid crops such as bread wheat (*Triticum aestivum*) and by aquatic species such as common carp (*Cyprinus carpio*) and gibel carp (*Carassius gibelio*) [4,5]. In breeding programs, artificially induced triploids and other polyploids are widely used to generate sterile stocks, preventing undesired reproduction in natural ecosystems and simultaneously improving production traits, including growth rate and flesh quality [6,7,8]. These successes underscore the key role of allopolyploidy in improving economic traits [9,10], and therefore, synthesizing novel sterile allopolyploids is of great value [11,12].

In allopolyploids, the ancestral contributor genomes are termed subgenomes, and their corresponding duplicated genes are known as homeologs. When divergent subgenomes merge within a shared nucleus, the genetic architecture, transcriptional networks, and epigenetic states will fundamentally reshape in allopolyploids relative to their diploid ancestors [13,14,15]. Analyzing transcriptomes in allopolyploids helps us understand homoeolog expression and how these allopolyploids acquire novel traits and adapt to new environments [9]. Additive expression is generally regarded as the expected baseline for homoeolog expression. Deviations from this expectation, collectively referred to as non-additive expression, include expression-level dominance, homoeolog expression bias, and transgressive expression and have been widely observed in many allopolyploid plants as important sources of regulatory innovation [16,17,18]. These plant studies have provided a useful framework for understanding how divergent subgenomes interact after allopolyploidization. Comparable studies in animals have revealed asymmetric subgenome evolution and divergent homoeolog expression in allotetraploid vertebrates, including *Xenopus laevis* and allotetraploid cyprinid fishes [19,20]. However, these studies have mainly focused on established polyploid lineages, whereas comparable multi-tissue analyses in newly synthesized animal allopolyploids remain limited. Newly synthesized allopolyploids provide useful systems for investigating early transcriptional changes associated with hybridization and polyploidization [21].

Grass carp (*Ctenopharyngodon idella*) is one of the most important freshwater aquaculture species globally (especially in China) [22]. It is known for very rapid growth compared to many other fish and valued both as a food fish and for its use in aquatic weed control [23,24]. Despite its economic importance, there is a notable lack of commercially viable polyploid grass carp varieties, particularly sterile lines that could facilitate genetic containment, reduce ecological risks associated with escapees, and prevent unwanted reproduction in aquaculture systems. Efforts such as inducing triploidy or creating interspecific hybrids have been made to try to boost growth and disease resistance in grass carp [25,26]. Polyploid grass carp have been explored, and autotriploid grass carp and their embryonic transcriptomic changes have been reported; however, synthetic allopolyploid grass carp and systematic analyses of their phenotypes and transcriptomes remain largely lacking.

Here, we synthesized a sterile allotriploid between grass carp as the maternal species and yellowcheek (*Elopichthys bambusa*) as the paternal species using an optimized hydrostatic pressure protocol. Yellowcheek was selected as the paternal species because it has the same chromosome number as grass carp and is phylogenetically closely related to it, yet the two species show contrasting feeding ecologies, with yellowcheek being piscivorous and grass carp herbivorous. This combination provides a suitable system for examining parental genome interactions between closely related but ecologically divergent species [27]. In this study, we aimed to (1) confirm the cytogenetic and genomic constitution of the newly synthesized grass carp × yellowcheek allotriploid using flow cytometry, chromosome counting, and whole-genome resequencing; (2) characterize expression-inheritance patterns across seven tissues; (3) quantify maternal and paternal genome contributions using expression-level dominance, allele-specific expression, and cis/trans regulatory analyses; and (4) integrate intestinal morphology and histology with the expression of genes related to digestion, nutrient transport, smooth muscle, and epithelial function.

## 2. Materials and Methods

### 2.1. Experimental Fish and Ethics Statement

The parental grass carp and yellowcheek and the induced triploid progeny were bred and maintained at the National Aquatic Biological Resource Center, Institute of Hydrobiology, Chinese Academy of Sciences, under standardized freshwater conditions. No privately owned or external farm-owned fish were used in this study. Before artificial propagation, broodstock were acclimated under the same freshwater conditions. All experimental procedures and fish handling were approved by the Animal Care and Use Committee of the Institute of Hydrobiology, Chinese Academy of Sciences (approval code: IHB/LL/2024047; approval date: 10 February 2024) and were conducted in strict accordance with the committee guidelines.

### 2.2. Artificial Insemination and Hydrostatic Pressure Treatment

Artificial insemination was performed using eggs collected from five female grass carp (*Ci*) and milt collected from five male yellowcheek (*Eb*). Prior to gamete collection, the genital area of the broodstock was dried to prevent contamination by water/urine. Milt was stored at 4–10 °C in the dark, while eggs were stripped into plastic basins. Artificial insemination was conducted via the dry method in a dimly lit environment, using 5 mL of milt per 100,000 eggs. Sperm was activated with fresh water and stirred gently with a feather to ensure contact. The mixture was stirred for another 0.5 min with additional water and then rinsed 2–3 times to remove excess sperm and debris.

Hydrostatic pressure treatment refers to exposing fertilized eggs to a controlled high-pressure pulse to inhibit second polar-body extrusion and retain an additional maternal chromosome set. Based on a previously reported protocol for grass carp [28], triploidy was induced using a hydrostatic pressure chamber at 23 ± 1 °C. Pressure intensity, treatment initiation time, and treatment duration were optimized for the present cross, and the final condition was selected by balancing triploidy rate with fertilization and hatching performance. The experiment evaluated three variables: initiation time (2, 2.5, and 3 min), pressure intensity (34, 48, and 68 MPa), and duration (1.5, 2, and 2.5 min). Initiation time was defined as the period between fertilization and the start of pressure treatment. At the designated times, fertilized eggs were placed in a steel sieve cylinder within the chamber. Pressure was increased uniformly to the target level, maintained for the specified duration, and then released to atmospheric pressure. For each treatment condition, pooled gametes from five pairs of broodstock were used, and 100 embryos were randomly examined to determine fertilization and hatching rates, while 100 larvae were randomly sampled to assess the triploidy rate. Because these optimization data were based on treatment-level counts rather than independent replicated batches, the rates are presented as descriptive percentages, and no inferential statistical test was applied for intergroup comparisons. Triploidy rate was assessed by flow cytometric analysis of nuclear DNA content, using diploid parental samples as controls.

### 2.3. Morphometric Measurements, DNA-Content Analysis, Chromosome Preparation, and Gonadal Examination

Eight-month-old *Ci*, CCE, and *Eb* individuals reared under the same conditions were used for morphometric, intestinal histological, and transcriptomic analyses. External morphology was recorded for randomly selected *Ci*, CCE, and *Eb* individuals using a digital camera under the same imaging conditions. Body weight (BW), total length (TL), body length (BL), head length (HL), and body height (BH) were measured in three individuals per group (*n* = 3). Relative body indices were calculated as BH/BL, BW/BL, and HL/BL. All measurements were performed using a ruler or digital caliper according to the same measurement criteria.

The DNA content of triploids was measured by flow cytometry (CytoFLEX S, Beckman Coulter, Inc., Brea, CA, USA). Following the methods of Yang et al. [29], blood samples were prepared with CyStain^®^ DNA 1 Step solution (Sysmex Partec GmbH, Görlitz, Germany), with diploid parental samples used as 2n controls. Subsequently, metaphase chromosomes were prepared from three randomly selected triploid individuals according to Li et al. [30] and Zhu and Gui [31]. For each individual, 20 high-quality metaphase spreads were examined to determine chromosome number.

For gonadal examination, gonads were dissected from one-year-old *Ci*, CCE, and *Eb* individuals and photographed. Because grass carp generally require 4–5 years to reach sexual maturity, the one-year-old individuals were examined only to assess gonadal developmental status at the sampled stage. Samples were fixed in 4% paraformaldehyde, dehydrated in a graded ethanol series, cleared in xylene, embedded in paraffin, sectioned at 5 µm, and stained with hematoxylin and eosin (H&E). Gonadal sections were examined under a microscope to assess gametogenic development.

### 2.4. Tissue Collection and Sampling Design

For transcriptome sequencing, seven tissues, including brain, hypothalamus, liver, gut, kidney, spleen, and muscle, were collected from eight-month-old *Ci*, CCE, and *Eb* individuals. For each group and tissue, three biological replicates were prepared. In total, 63 RNA-seq libraries were constructed and sequenced, corresponding to three groups, seven tissues, and three biological replicates per tissue. At the sampling stage, sex could not be reliably determined by external morphology and was therefore not controlled, as also reported in a transcriptomic study of juvenile Atlantic salmon [32]. All tissues were rapidly dissected, immediately frozen in liquid nitrogen, and stored at −80 °C until RNA extraction. For qPCR validation analyses, intestinal samples were collected from three biological replicates per group.

### 2.5. Intestinal Morphology and Histological Analysis

For gross intestinal morphology, the entire intestine was dissected from *Ci*, CCE, and *Eb* individuals. Intestinal length (IL) was measured using a ruler after gently straightening the intestine without stretching. Relative intestinal length was calculated as IL/TL, where TL represents total body length. For each group, three individuals were measured. For histological analysis, mid-intestine segments were collected consistently from all individuals and fixed in 4% paraformaldehyde, dehydrated through a graded ethanol series, cleared in xylene, and embedded in paraffin. Paraffin blocks were sectioned at 5 μm using a Leica RM2235 rotary microtome (Leica Biosystems Nussloch GmbH, Nussloch, Germany). H&E-stained sections were scanned using a KF-PRX-040 bright-field whole-slide scanner (Ningbo Jiangfeng BioInformation Technology Co., Ltd., Ningbo, China). Representative images were captured at 4× magnification for overall intestinal morphology and at 20× or 80× magnification for local mucosal structure. Villus height, muscularis thickness, and goblet cell abundance were quantified using ImageJ software version 1.53t. Goblet cells were manually counted along both visible sides of one well-oriented intestinal villus/fold per sample based on their pale, vacuole-like morphology in H&E-stained sections. The results are expressed as the number of goblet cells per villus/fold.

### 2.6. Whole-Genome Resequencing and Genomic Constitution Analysis

Genomic DNA was extracted from fin tissues of representative *Ci*, CCE, and *Eb* individuals using a DNA extraction kit (Sangon Biotech Co., Ltd., Shanghai, China) following the manufacturer’s protocol. Briefly, fin tissues were digested in lysis buffer containing proteinase K until sufficiently lysed. The lysate was then applied to a spin column, washed with the supplied wash buffers, and genomic DNA was eluted with ddH_2_O. DNA quality and concentration were assessed using a NanoDrop spectrophotometer(Thermo Fisher Scientific, Waltham, MA, USA). Whole-genome resequencing libraries were constructed following the manufacturer’s protocol and sequenced on an Illumina platform (Illumina, Inc., San Diego, CA, USA) to generate paired-end reads. The average sequencing depth across all samples was approximately 10×.

To analyze the genomic constitution of CCE, a composite reference genome was constructed by concatenating the chromosome-level genome assemblies of *Ci* [33] and *Eb* (GenBank accession: GCA_037101425.1). Clean reads from *Ci*, CCE, and *Eb* were independently mapped to the composite reference using BWA-MEM. SAMtools (v 1.15.1) was used to sort alignments, remove low-quality alignments, and calculate read depth across chromosomes [34]. Read-depth profiles were plotted to compare the relative coverage of maternal and paternal genomic regions. The maternal-to-paternal coverage ratio was estimated based on genome-wide average depth across the *Ci*-derived and *Eb*-derived regions.

### 2.7. RNA Extraction, Library Preparation, and Sequencing

Total RNA was extracted from the tissues stored at −80 °C using TRIzol reagent (Invitrogen, Carlsbad, CA, USA). The purity and integrity of the RNA were assessed using a NanoDrop spectrophotometer and an Agilent 2100 Bioanalyzer (Agilent Technologies, Santa Clara, CA, USA). Only RNA samples with RNA integrity number (RIN) values greater than 7.0 were used for library construction. mRNA was enriched from total RNA using kits with Oligo(dT) magnetic beads and then fragmented. The fragmented mRNA served as a template for first-strand cDNA synthesis via reverse transcription, followed by second-strand cDNA synthesis. The purified double-stranded cDNA underwent end-repair, A-tailing, and ligation of sequencing adapters. The final cDNA libraries were constructed through PCR amplification. After quality control with Qubit and the Agilent 2100, the libraries were sequenced on an Illumina NovaSeq platform to generate 150 bp paired-end reads. Across the 63 RNA-seq libraries, each library generated an average of 68.96 million clean reads.

### 2.8. RNA-Seq Data Processing, Differential Expression Analysis, and Expression-Level Dominance Classification

The quality of the raw sequencing reads was assessed using FastQC. Adapter sequences and low-quality reads were removed using Trimmomatic to obtain high-quality clean reads. The clean reads from the parents (*Ci*, *Eb*) and the triploids (CCE) were aligned to the concatenated *Ci*-*Eb* composite reference genome (BioProject accession: PRJNA745929 and GCA_037101425.1) using HISAT2. Reads were aligned to a concatenated parental reference containing both maternal and paternal genomic sequences, and only orthologous/homeologous gene pairs with reliable one-to-one correspondence were retained for downstream expression comparison. The R package DESeq2 was used to normalize the read count matrix and perform differential expression analysis. To determine the inheritance patterns of DEGs, we employed the classification framework developed by Rapp et al. [35] as implemented in the R package HybridExpress [36]. Differential expression analyses were performed separately for each tissue using group as the main factor, without including batch effects due to uniform library preparation and sequencing workflow.

Expression-level dominance and non-additive expression patterns were analyzed using the HybridExpress workflow following Almeida-Silva et al. [36], which implements the expression classification framework of Rapp et al. [35]. The mid-parent value (MPV) was calculated for each gene as the arithmetic mean expression level of the two parents, *Ci* and *Eb*. For each tissue, gene expression in CCE was compared with that in the two parents. Four contrasts were tested using DESeq2: *Eb* vs. *Ci*, CCE vs. *Ci*, CCE vs. *Eb*, and CCE vs. MPV. Genes were classified into expression categories based on the direction and significance of these contrasts.

The 12 expression categories generated by HybridExpress were further summarized into five major expression classes: additive expression (ADD), expression-level dominance toward *Ci* (ELD-*Ci*), expression-level dominance toward *Eb* (ELD-*Eb*), transgressive upregulation (UP), and transgressive downregulation (DOWN). Classification was based on pairwise comparisons among *Ci*, *Eb*, CCE, and the MPV, using FDR < 0.05 and |log2 fold change| ≥ 1 as significance thresholds. Briefly, ADD genes showed no significant difference between CCE and MPV and were intermediate between the two parents; ELD-*Ci* and ELD-*Eb* genes showed no significant difference between CCE and the corresponding dominant parent but differed from the other parent; UP and DOWN genes were expressed significantly higher or lower in CCE than in both parents, respectively. ELD-*Ci* genes were defined as genes for which CCE expression was statistically closer to *Ci* than to *Eb*, whereas ELD-*Eb* genes were defined as genes for which CCE expression was statistically closer to *Eb* than to *Ci*. UP and DOWN genes were defined as genes whose expression in CCE was higher or lower, respectively, than both parents. Differential expression was considered significant at an adjusted value (FDR) < 0.05 and |log2 fold change| ≥ 1.

For ELD-*Eb* genes, inherited dominance was further assessed by comparing parental expression levels. An ELD-*Eb* gene was considered to show inherited paternal dominance when its expression in *Eb* was higher than in *Ci* in the parental comparison. ELD-*Eb* genes without higher parental expression in *Eb* were classified as novel paternal dominance in the triploid. The proportion of inherited and novel ELD-*Eb* genes was calculated separately for each tissue.

### 2.9. Allele-Specific Expression and Cis/Trans Regulatory Classification

For ASE analysis, a database of single-nucleotide polymorphism (SNP) sites capable of distinguishing the two parental origins was first constructed using parental genomic sequencing data. RNA-Seq reads from the triploids were then aligned to a reference genome containing this SNP information. Allelic read counts supporting *Ci*- and *Eb*-derived alleles were extracted using ASEReadCounter (GATK v4.2.6.1) [37].

For each gene, allelic read counts were summed across informative SNPs. Informative SNPs were defined as sites that were fixed for different alleles between the two parents. Genes were retained for ASE analysis only when they contained informative SNPs and had at least 20 total allelic reads summed across these SNPs. A binomial test was used to assess whether the observed *Eb*:*Ci* allelic expression ratio deviated from the expected 1:2 ratio based on the genomic dosage of CCE. The overall *cis*- and trans-regulatory effects were inferred by comparing parental expression divergence between *Ci* and *Eb* with allelic expression divergence between *Ci*- and *Eb*-derived alleles in CCE, following the framework of Wittkopp et al. [38]. Genes were assigned to six categories: *cis*-only, trans-only, *cis* + *trans*, compensatory, conserved, and ambiguous. Genes with insufficient informative SNPs or low read support were not classified.

### 2.10. Gut-Related Gene Heatmap and qPCR Validation

For visualization of intestinal gene-expression patterns, genes shown in the heatmap were selected based on functional annotation and published roles in intestinal functions, including smooth muscle structure and contraction, protein digestion, lipid digestion and absorption, carbohydrate digestion and absorption, amino acid/peptide transport, and epithelial/barrier function. For each selected gene, group mean FPKM values from three biological replicates were calculated, transformed as log2(FPKM + 1), row-scaled by Z-score normalization, and visualized as a heatmap. Genes were manually ordered by functional category to facilitate comparison among *Ci*, *CCE*, and *Eb*.

First-strand cDNA synthesized from intestinal RNA samples was used as the template for qPCR validation. qPCR was performed using gene-specific primers for *myh14*, *smtnl1*, *muc5e*, and *slc26a2* (Table S1). The internal reference gene was *β-actin*. Relative expression levels were calculated using the 2^−ΔΔCt^ method. Three biological replicates were analyzed for each group, and each reaction included three technical replicates.

### 2.11. Statistical Analysis

Data from morphometric and histological measurements are presented as mean ± SD, whereas qPCR data are presented as mean ± SEM. Values in Table 1 and Table 2 are presented as descriptive percentages calculated from embryo or larval counts. For RNA-seq data, *p*-values were adjusted using the Benjamini–Hochberg false discovery rate correction. For other datasets, normality was assessed using the Shapiro–Wilk test, and homogeneity of variance was evaluated using Levene’s or Brown–Forsythe tests before ANOVA. Statistical differences among groups were analyzed using one-way ANOVA followed by Tukey’s multiple-comparison test. For morphometric, histological, and qPCR analyses, n represents biological replicates unless otherwise stated. Statistical analyses were performed using GraphPad Prism 8.0 (version 8.0.0).

## 3. Results

### 3.1. Optimizing Conditions of Triploidy Induction in Grass Carp

To determine the optimal conditions for inducing triploidy of grass carp, we systematically evaluated the effects of hydrostatic pressure on fertilization, hatching, and triploidy rates. With a fixed shock duration of 2 min, we tested different pressure intensities and shock initiation timings. A clear pressure-dependent effect was observed: a low pressure of 34 MPa produced a triploidy rate of 80%, whereas a high pressure of 68 MPa achieved 100% triploidy but severely reduced hatching rates to only 3–5%. By contrast, 48 MPa also achieved 100% triploidy while maintaining substantially higher fertilization and hatching rates and was therefore selected for subsequent optimization (Table 1).

At 48 MPa, we compared two shock initiation timings. Shock applied at 3.5 min post-fertilization resulted in a slightly higher fertilization rate (96%) than at 2.5 min (92%), while hatching rates were similar (70% vs. 71%; Table 1). Therefore, a pressure of 48 MPa applied for 2 min beginning at 3.5 min post-fertilization was selected as the optimal condition for further refinement.

We further examined the effect of treatment duration (1.5, 2, and 2.5 min) under the selected pressure condition (Table 2). Triploidy rates remained 100% across all tested durations, and the fertilization rate was stable at 96%. However, hatching rates declined with longer treatment: hatching rates for the 1.5 min and 2 min treatments were comparable (72% and 70%, respectively), whereas the lowest hatching rate (66%) occurred at 2.5 min. These results indicate that prolonging the shock beyond 2 min reduces survival without improving induction efficiency.

Altogether, the optimal triploidy induction conditions for grass carp were determined to be a hydrostatic pressure of 48 MPa applied for 1.5 to 2 min, with shock initiation at 3.5 min post-fertilization. This combination ensures 100% triploidy while avoiding the high mortality associated with higher pressure (68 MPa) or longer treatment (2.5 min), achieving the best balance between induction efficiency and embryo survival.

### 3.2. Genetic Identification of Triploid Progeny

**Figure 1:**
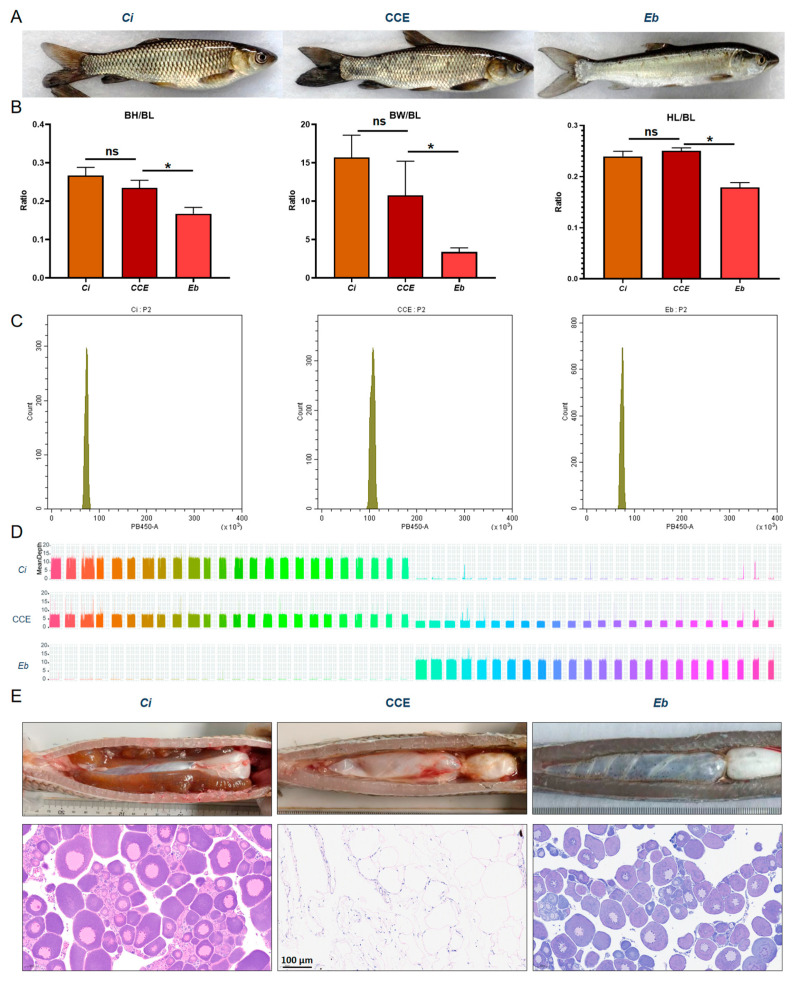
(**A**) External morphology of the maternal parent *Ci*, triploid CCE, and paternal parent *Eb*. (**B**) Body-shape indices, including body height/body length (BH/BL), body weight/body length (BW/BL), and head length/body length (HL/BL). Data are presented as mean ± SD; ns, not significant; * *p* < 0.05. (**C**) Flow cytometric analysis of erythrocyte nuclear DNA content. (**D**) Whole-genome resequencing read-depth profiles mapped to the combined parental reference genome, showing an approximately 2:1 maternal-to-paternal genomic ratio in CCE. Different colors are used to distinguish different chromosomes. (**E**) Gross gonadal morphology and H&E-stained gonadal sections of *Ci*, CCE, and *Eb*. Scale bar = 100 μm.

To verify the successful induction and characterize the genetic origin of the progeny (designated CCE), we performed a multifaceted analysis. First, morphological examination revealed that the CCE individuals exhibited intermediate traits, but overall body conformation more closely resembled the maternal grass carp (Figure 1A). Quantitative comparisons further supported this pattern. For BH/BL, BW/BL, and HL/BL, CCE was not significantly different from *Ci* (*p* = 0.1404, 0.0940, and 0.3298, respectively) but differed significantly from *Eb* (*p* = 0.0016, 0.0067, and <0.0001, respectively), indicating a predominantly maternal-like body conformation. Next, flow cytometry analysis showed that both parents had diploid DNA content, whereas the CCE group displayed a fluorescence peak approximately 1.5 times higher (Figure 1C), consistent with triploidy. Third, karyotyping analysis showed that somatic cells of CCE individuals contained 72 chromosomes (3n) (Figure S1), in contrast to the 48 chromosomes (2n) observed in both parents [27,39]. Finally, to verify the genomic composition of the hybrid, we carried out whole-genome resequencing using a combined reference built from the maternal (*Ci*) and paternal (*Eb*) genomes. As expected, reads from each parent mapped predominantly to their own genome, confirming the specificity of the reference. In contrast, reads from the CCE progeny aligned to both parental genomes, demonstrating the presence of genomic contributions from both species (Figure 1D). Quantitative coverage analysis further showed that the depth across maternal genomic regions was approximately twice that across paternal regions, consistent with the expected 2:1 maternal–paternal genomic dosage of the triploid hybrid. In addition, gross gonadal observation and histological examination revealed clear differences among the three groups. Gonads were dissected from one-year-old *Ci*, CCE, and *Eb* individuals. *Ci* and *Eb* displayed normal tissue, whereas CCE showed markedly reduced and abnormal gonadal tissue with reduced gonadal size, poorly developed germinal tissue, and absence of obvious mature gametogenic structures (Figure 1E), consistent with its sterile phenotype. Collectively, these results support the conclusion that CCE is a sterile triploid hybrid containing two maternal and one paternal haploid chromosome sets.

### 3.3. Parental Bias and Tissue-Dependent Regulatory Divergence in Triploid CCE

**Figure 2:**
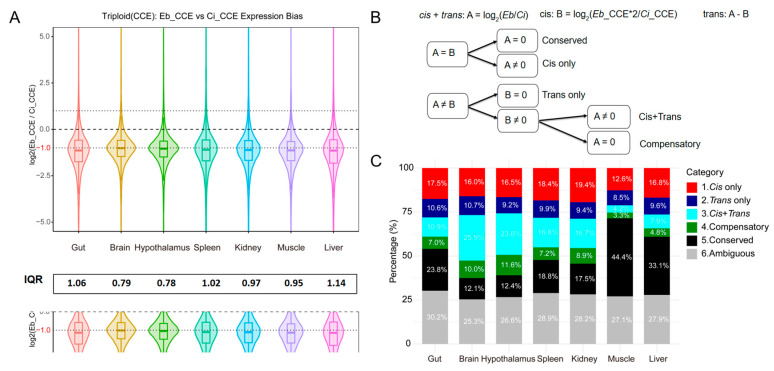
Tissue-specific allelic expression bias and regulatory modes in triploid CCE: (**A**) Violin plots showing the distribution of homeolog expression bias log2(*Eb*_CCE/*Ci*_CCE) across seven tissues. The dashed line at 0 indicates balanced expression. Internal boxplots represent the median and quartiles. The table below lists the Interquartile Range (IQR) values for each tissue, indicating the variability of expression bias. (**B**) Schematic diagram illustrating the decision tree used to classify regulatory divergence categories. Classification is based on the comparison between total parental expression divergence and the allele-specific expression ratio in the triploid. Genes are categorized into Conserved, *Cis*-only, *Trans*-only, *Cis* + *Trans*, and Compensatory. (**C**) Stacked bar charts displaying the percentage composition of genes assigned to each regulatory category across the seven tissues. Categories are color-coded as indicated in the legend on the right.

To explore how the combination of divergent parental subgenomes shapes gene expression in triploid CCE, we performed allele-specific expression (ASE) analysis across seven tissues. By quantifying and comparing the expression of paternal (*Eb*) and maternal (*Ci*) alleles (Table S2), we found that the expression ratio across all tissues was centered around log2(*Eb*/*Ci*) = −1, corresponding to a 1:2 (*Eb*:*Ci*) expression ratio, consistent with the expected genomic dosage. This matches the expected 1:2 genomic dosage (one *Eb* and two *Ci* allele copies), indicating that overall expression follows gene copy number. Although all tissues conformed to the dosage pattern, the degree of variation around the expected ratio differed among tissues. Violin plots revealed that the brain and hypothalamus exhibited a narrower distribution of expression ratios around the median compared to other tissues. This was supported by interquartile range (IQR) analysis: the brain and hypothalamus had the lowest IQR values (0.79 and 0.78, respectively), in contrast to the higher dispersion seen in the liver (IQR = 1.14) and gut (IQR = 1.06) (Figure 2A). The lower dispersion of *Eb*:*Ci* allelic expression ratios observed in neural tissues is consistent with tighter maintenance of dosage-balanced allelic expression, whereas metabolic tissues showed greater variation around the expected ratio.

To understand the regulatory basis of these expression patterns, we decomposed divergence into *cis*- and *trans*-regulatory effects (Figure 2B). Genes were classified based on their allelic expression patterns relative to parental divergence (Figure 2C). A substantial proportion of genes showed “Conserved” or “Ambiguous” regulation across tissues (e.g., 44.4% conserved in muscle), reflecting widespread regulatory stability. Among genes with divergent expression, “*Cis*-only” effects were predominant in most tissues (ranging from 12.6% in muscle to 19.4% in kidney), suggesting that *cis*-regulatory variation contributes substantially to expression differences between subgenomes. Notably, tissue-specific regulatory landscapes were apparent in the distribution of complex regulatory categories. Neural tissues (brain and hypothalamus) were distinguished by high proportions of genes under combined “*Cis* + *Trans*” regulation (25.9% and 23.6%, respectively), significantly higher than in muscle (4.2%) or liver (7.9%). This suggests that in neural tissues, *cis*- and trans-regulatory changes often co-occur, potentially compounding expression divergence. Conversely, muscle displayed high conservation and minimal complex regulation, consistent with more constrained regulation in muscle. These results suggest that while gene expression in triploid CCE broadly reflects the 2:1 maternal genomic dosage, the underlying regulatory divergence varies markedly across tissues.

### 3.4. Tissue-Specific Inheritance Patterns and Parental Expression Dominance in Triploid CCE

**Figure 3:**
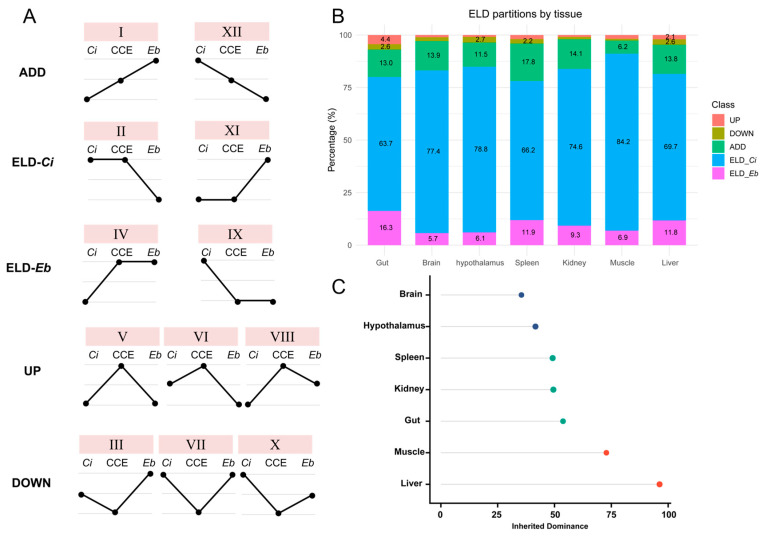
Classification and landscape of non-additive gene expression patterns in triploid CCE: (**A**) A schematic diagram illustrating the classification of gene expression patterns. Genes were classified into five categories based on the expression level of the triploid (CCE) relative to the two parents (*Ci*, *Eb*): additive (ADD), expression level dominance of the *Ci* parent (ELD-*Ci*), expression level dominance of the *Eb* parent (ELD-*Eb*), transgressive upregulation (UP), and transgressive downregulation (DOWN). (**B**) Percentage distribution of genes for each pattern across different tissues. The stacked bar chart shows the proportion of genes belonging to the five inheritance patterns defined in (**A**) for each of the seven tissues. (**C**) Tissue-specific ranking of inherited dominance.

To determine the overall patterns of expression inheritance in triploid CCE, we compared its total gene expression levels with those of its parents. Genes were classified into five categories based on their relative expression: additive (ADD, intermediate between parents), expression level dominance biased toward either the maternal (ELD-*Ci*) or paternal (ELD-*Eb*) parent, and transgressive up- or downregulated (UP/DOWN) relative to both parents (Figure 3A). Detailed gene lists, expression classes, and category assignments for the seven tissues are provided in Tables S3–S9. Expression level dominance was the predominant inheritance pattern across all tissues, with the majority of differentially expressed genes showing a parental bias rather than an intermediate additive pattern (Figure 3B). Maternal dominant (ELD-*Ci*) was particularly widespread, accounting for 63.7% to 84.2% of classifiable transcripts, consistent with the 2:1 maternal-to-paternal genomic dosage and reflecting dosage-associated maternal-biased expression in most genes.

Despite this global maternal background, a subset of genes exhibited paternal dominance (ELD-*Eb*). This analysis revealed striking tissue-specific differences in the origin of paternal dominance (Figure 3C). Liver and muscle displayed high inherited dominance, with 96.2% and 72.8% of paternally dominant genes, respectively, reflecting a pre-existing paternal high-expression state. Gut (53.2%), kidney (49.5%), and spleen (49.2%) showed intermediate inheritance. In contrast, neural tissues (brain 35.4%; hypothalamus 41.6%) were dominated by “Novel” dominance, defined as ELD-*Eb* genes that do not show pre-existing higher expression in *Eb* relative to *Ci* in the parental comparison. The novel paternal dominance may indicate new regulatory states after hybridization, including paternal allele activation, maternal allele suppression, or both. KEGG enrichment analysis showed that novel ELD-*Eb* genes in the brain and hypothalamus were enriched in pathways related to cell adhesion, cytoskeleton regulation, intercellular communication, and nucleotide metabolism, including focal adhesion, ECM-receptor interaction, regulation of actin cytoskeleton, gap junction, purine metabolism, and nucleotide metabolism (Figure S2).

We further investigated the biological roles of those paternally dominant genes across tissues with contrasting inheritance profiles. In the liver, which exhibited the highest inherited dominance, the triploid retained paternal-biased expression of genes involved in hematopoiesis and coagulation, including thrombopoietin (a key regulator of platelet production), trypsinogen (a digestive enzyme precursor involved in protein digestion), and GRIN2A (a subunit of NMDA receptors associated with synaptic signaling), as well as carboxypeptidase B2 (Table S6). In the gut, an intermediate-inheritance tissue, key genes associated with protein digestion (*trypsinogen*, *tmprss15*) and peptide transport (*slc15a1*) showed strong paternal-biased expression (Table S3). In the brain, the tissue with the highest proportion of novel dominance, paternal-biased expression was observed for genes involved in synaptic signaling and excitatory–inhibitory balance (e.g., *NMDA receptor grin2a*, *GABA receptors*; Table S4), representing expression patterns not predictable from either parental species alone.

In summary, the triploid transcriptome is shaped by a dual regulatory architecture. Globally, the 2:1 genomic dosage imposes a maternal-like expression landscape, contributing to transcriptomic stability. Locally, however, the paternal subgenome exerts tissue-specific influence. This demonstrates that gene expression in triploids is not a simple dosage-scaled average of parental programs but involves tissue-dependent contributions from each parental genome, providing candidate molecular substrates underlying the observed phenotypic differences between the triploid and its parental species.

### 3.5. Gut Morphological Differences in CCE Coincide with Distinct Gene Expression Patterns

**Figure 4:**
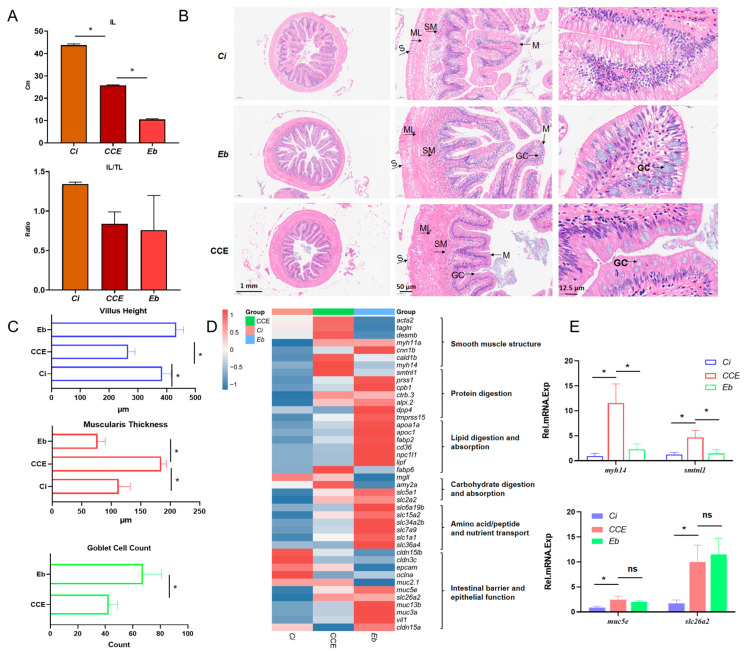
Integrated morphological and transcriptional analyses reveal distinct gut traits and gene expression patterns in triploid CCE: (**A**) Gross intestinal phenotypes of the maternal parent (*Ci*), the triploid (CCE), and the paternal parent (*Eb*). Upper panel, intestinal length (IL); lower panel, relative intestinal length (IL/TL). (**B**) H&E-stained gut sections at low (4×), intermediate (20×), and high magnification (80×); arrows indicate representative goblet cells. Scale bars = 1 mm, 50 μm, and 12.5 μm, respectively. ML (muscularis layer), SM (submucosa), S (serosa), GC (goblet cells), M (mucus). (**C**) Morphometric analysis of villus height, muscularis thickness, and goblet cell abundance, expressed as goblet cells per villus/fold. Data are presented as mean ± SD. (**D**) Heatmap showing group mean expression profiles of selected genes associated with smooth muscle structure, protein digestion, lipid digestion and absorption, carbohydrate digestion and absorption, amino acid/peptide and nutrient transport, and intestinal barrier and epithelial function. (**E**) qPCR validation of representative genes associated with smooth muscle structure (*myh14*, *smtnl1*) and epithelial/mucus-related function (*muc5e*, *slc26a2*). Data are presented as mean ± SEM. * *p* < 0.05; ns, not significant.

Given the pronounced paternal-biased expression (ELD-*Eb*) observed in the gut, we further examined this organ at the morphological, histological, and transcriptional levels in CCE and its parents (Figure 4). At gross morphology, intestinal length (IL) differed markedly among groups, being longest in *Ci*, intermediate in CCE, and shortest in *Eb* (Figure 4A, upper; all pairwise comparisons, *p* < 0.0001). After normalization to total body length (TL), relative intestinal length (IL/TL) was highest in *Ci*, intermediate in CCE, and lowest in *Eb* (Figure 4A, lower; *Ci* vs. CCE, *p* = 0.000052; *Ci* vs. *Eb*, *p* < 0.000001; CCE vs. *Eb*, *p* = 0.000173). Thus, CCE showed an intermediate intestinal-length phenotype, with relative intestinal length closer to *Eb* than to *Ci*.

H&E staining showed that all three groups retained the typical four-layer intestinal structure, including serosa (S), muscularis layer (ML), submucosa (SM), and mucosa (Figure 4B). However, morphometric quantification revealed clear differences in fine structure (Figure 4C). Villus height was greatest in *Eb* (~430 μm), intermediate in *Ci* (~380 μm), and lowest in CCE (~260 μm), with CCE showing significantly shorter villi than both parents (CCE vs. *Ci*, *p* = 0.000036; CCE vs. *Eb*, *p* = 0.000001). Muscularis thickness showed the opposite pattern: it was highest in CCE (~185 μm), exceeding that of both *Ci* (~110 μm; *p* = 0.000022) and *Eb* (~75 μm; *p* < 0.000001). Goblet cells were clearly identifiable in *Eb* and CCE but were not consistently distinguishable in *Ci* sections under H&E staining; therefore, quantitative comparison was limited to CCE and *Eb*. Goblet cell abundance was lower in CCE than in *Eb* (*p* = 0.0452). Thus, the CCE intestine combined shorter villi, a thicker muscularis layer than both parents, and reduced goblet cell abundance relative to *Eb*.

To examine whether these morphological features coincided with distinct expression profiles, we inspected group-level expression of genes assigned to functional categories related to gut structure and digestive/absorptive function (Figure 4D). First, smooth muscle-related genes (e.g., *acta2*, *tagln*, *desmb*, *myh11a*, *cnn1b*, *cald1b*, and *myh14*) were generally more highly expressed in CCE than in either parent, paralleling the thicker muscularis observed histologically. Second, genes involved in digestion, absorption, and nutrient transport—including those related to protein digestion, lipid digestion and absorption, and amino acid and peptide transport, as well as most carbohydrate-related genes (*amy2a*, *slc5a1*, and *slc2a2*)—were more highly expressed in *Eb* than in *Ci*, and this *Eb*-like profile was largely retained in CCE, consistent with the prominent ELD-*Eb* signal previously detected in this tissue. Genes related to intestinal barrier and epithelial function showed a more heterogeneous pattern. Tight junction-associated genes (*cldn15lb*, *cldn3c*, *oclna*, and *epcam*) were generally higher in *Ci*, while several mucin genes and related transporters (*muc2*.1, *muc5e*, *slc26a2*, *muc13b*, *muc3a*, *vil1*, and *cldn15a*) were higher in *Eb* or in both *Eb* and CCE. This heterogeneity within a single functional category indicates that not all gut-related gene groups follow a simple inherited pattern in CCE.

The smooth muscle-related genes myh14 and smtnl1 were significantly more highly expressed in CCE than in either parent, representing non-additive expression beyond the parental range (*myh14*: CCE vs. *Ci*, *p* = 0.0040; CCE vs. *Eb*, *p* = 0.0073; *smtnl1*: CCE vs. *Ci*, *p* = 0.0114; CCE vs. *Eb*, *p* = 0.0151). In contrast, the epithelial/mucus-related genes *muc5e* and *slc26a2* were significantly higher in CCE than in *Ci* but not significantly different from *Eb*, consistent with an *Eb*-like paternal-biased expression pattern (*muc5e*: CCE vs. *Ci*, *p* = 0.0119; CCE vs. *Eb*, *p* = 0.4946; *slc26a2*: CCE vs. *Ci*, *p* = 0.0224; CCE vs. *Eb*, *p* = 0.7753).

Together, these results show that the CCE intestine displays a combination of inherited and non-additive features at both the morphological and transcriptional levels: digestion- and most epithelial mucus-related genes largely follow *Eb*-like expression, while smooth muscle-related genes show non-additive upregulation that co-occurs with a thickened muscularis layer; tight junction-related genes, however, do not follow this pattern.

## 4. Discussion

The triploid hybrid between grass carp and yellowcheek provides a useful experimental system for examining the immediate transcriptomic consequences of allopolyploid formation in vertebrates. One distinctive feature of this system is that the two parental species differ markedly in feeding ecology. Grass carp is an obligate herbivore, whereas yellowcheek is a piscivorous predator; therefore, genome merger in CCE brings together two subgenomes with divergent physiological and morphological backgrounds. Importantly, unlike autopolyploid systems generated through genome duplication within a single species, this model represents an allopolyploid context in which two evolutionarily and ecologically distinct genomes are combined. Based on this system, our study addresses three related aspects: the methodological basis for generating the material, the regulatory features of its transcriptomic architecture, and the gut as a tissue-level case study at the interface of the parents’ contrasting diets. 

### 4.1. Induction Efficiency and a Robust Protocol for Sterile Triploid Production

Our optimized protocol, consisting of 48 MPa hydrostatic pressure applied at 3.5 min post-fertilization for 1.5–2 min, reliably yielded 100% triploidy while maintaining high embryonic viability (Table 1 and Table 2). This efficiency is consistent with values reported for hydrostatic-pressure-based induction protocols, which have achieved triploid rates of 97–98% in previous studies [28,40]. In contrast, cold-shock methods reported in the literature generally yield lower triploid induction efficiencies (approximately 70%), although these values vary depending on species, developmental stage, and treatment conditions [25]. The resulting triploids are sterile, a trait of considerable value for aquaculture management by preventing uncontrolled reproduction and mitigating ecological risks [11]. Together, this protocol provides a methodological reference for sterile triploid production. However, laboratory induction and rearing conditions differ substantially from commercial farming systems, and large-scale application in grass carp aquaculture will require further validation of survival, growth performance, welfare, and other production-related traits under farming conditions.

### 4.2. Global Maternal Dominance: A Consequence of Genomic Dosage

At the genome-wide level, CCE exhibited profound maternal expression-level dominance (ELD-*Ci*), mirroring its 2:1 maternal-to-paternal genomic composition (Figure 2). This pattern is consistent with a passive, dosage-dependent mode of transcriptomic regulation, in which the numerical predominance of maternal alleles shapes the overall expression landscape and contributes to transcriptomic stability. Such dosage-associated maternal bias may be consistent with the morphological resemblance of CCE to its maternal parent, providing a possible explanatory context for the frequently reported maternal influence on hybrid phenotypes, although it does not establish a causal relationship between gene expression dominance and morphology.

Similar dosage-associated maternal expression biases have been reported in other polyploid fish systems. Transcriptome analyses of hybrid and triploid cyprinids consistently reveal preferential maternal expression, often accompanied by partial dosage compensation, a regulatory buffering process that reduces the expected increase in gene expression resulting from elevated gene copy number, thereby mitigating excessive dosage effects [41,42,43]. Comparable regulatory trends have also been observed in naturally occurring triploid fish complexes, where previous studies reported maternal-biased expression together with dosage compensation-like regulation that attenuates the effects of increased gene copy number in allopolyploid genomes [44], for example, in triploid fish systems showing buffered expression levels relative to the expected additive dosage. Collectively, these observations are consistent with an emerging pattern in which maternal genomic dosage establishes a stable transcriptional baseline in newly formed polyploids.

### 4.3. Tissue-Specific Paternal Contributions and Neural Susceptibility to Regulatory Change

Despite the overarching maternal dominance, the paternal subgenome was neither silent nor passively diluted. Instead, it contributes in a highly tissue-specific manner (Figure 3). The patterns governing paternal allele expression fall along a spectrum from strict inheritance to novel expression states. Metabolic and structural tissues such as liver and muscle predominantly exhibit “inherited” paternal dominance, retaining pre-existing high-expression programs crucial for core physiology (e.g., hematopoiesis, digestion). In contrast, neural tissues display a high degree of “novel” paternal dominance, which could not be explained by parental expression differences, together with a higher fraction of genes classified under combined *cis* + *trans* regulation.

Several non-exclusive factors may contribute to this apparent susceptibility of neural tissues. First, neural regulatory networks depend on extensive combinatorial transcription factor binding and long-range chromatin interactions [38] and may be intrinsically more sensitive to the altered trans-regulatory environment created by genome merger; the elevated *cis* + *trans* fraction we observed suggests that both allele-linked *cis*-regulatory divergence and changes in the shared trans-regulatory milieu contribute to neural expression divergence, consistent with simultaneous perturbation of multiple regulatory layers. Second, neural tissues typically express a larger and more heterogeneous gene complement, and we cannot fully exclude residual contributions from low-expression genes, reference mapping, or annotation biases. Third, because grass carp and yellowcheek differ in foraging and predatory behavior, their neural regulatory histories may be particularly prone to incomplete reconciliation in a newly formed triploid, although this interpretation remains speculative; the enrichment of novel paternal-biased expression in synaptic signaling genes, including NMDA and GABA receptor components—key regulators of excitatory and inhibitory neural transmission, respectively—is compatible with this view, but our data do not demonstrate circuit- or behavior-level consequences. Mechanistically, novel paternal dominance in neural tissues may reflect paternal allele activation, maternal allele suppression, or both, and distinguishing these alternatives will require allele-resolved analyses in future studies.

More broadly, such asymmetric contributions of parental subgenomes are increasingly recognized as a general feature of hybrid and polyploid transcriptomes, reflecting a balance between dosage-driven stability and tissue-specific regulatory perturbation [45,46,47].

### 4.4. Gut Morphological Divergence Is Accompanied by Distinct Expression Patterns

The intestinal morphological differences in CCE were quantitatively pronounced. Mean villus height was approximately 260 μm, representing reductions of 32% relative to *Ci* (~380 μm) and 40% relative to *Eb* (~430 μm). For comparison, dietary supplementation in red tilapia increased midgut and hindgut villus height by approximately 66% and 40%, respectively, together with improved growth and feed utilization [48], indicating that villus changes of this magnitude may have biological relevance. Mean muscularis thickness in CCE was approximately 185 μm, which was 68% greater than that in *Ci* (~110 μm) and 147% greater than that in *Eb* (~75 μm). This marked thickening, together with the non-additive upregulation of *myh14* and *smtnl1*, suggests remodeling of the intestinal smooth muscle compartment, although intestinal motility was not directly measured. Goblet cell abundance was also lower in CCE than in *Eb*, but this result should be interpreted cautiously because the cells were identified morphologically using H&E staining rather than mucin-specific staining [49].

The gut of CCE provides a tissue-level example in which inherited paternal expression, non-additive regulation, and morphological divergence occur together. First, *Eb*-like expression of digestion-, absorption-, and nutrient transport-related genes may reflect inherited paternal digestive programs from the piscivorous yellowcheek, although its effect on digestive performance remains unknown. Second, non-additive upregulation of smooth muscle-related genes, together with the thicker muscularis layer, suggests regulatory remodeling of intestinal muscle development or motility after hybridization and triploidization. Together, these findings suggest that the intestinal phenotype of CCE may involve both inherited paternal-like digestive expression and non-additive regulation of smooth muscle-related genes, although their functional consequences require further validation.

Distinguishing among these possibilities will require controlled feeding trials under matched diets, longitudinal sampling across developmental stages, functional assays of intestinal motility and digestive capacity, and cross-family replicates to assess reproducibility. Because goblet cells were identified based on H&E morphology and were not consistently distinguishable in *Ci*, conclusions regarding goblet cell abundance were limited to the comparison between CCE and *Eb*. Although similar sample sizes have been used in fish intestinal histology studies [50], the external morphometric and intestinal histological comparisons in the present study were based on three individuals per group; therefore, these findings should be interpreted with caution and confirmed using larger sample sizes. In addition, because sex was not controlled at the sampled stage, potential effects of sexual dimorphism on tissue-level expression patterns cannot be excluded. These considerations echo a recurring theme in the hybrid and allopolyploid literature: tissue-level phenotypes in newly formed genomic combinations are neither simple averages of parental traits nor necessarily adaptive novelties but may instead reflect the unresolved regulatory state of a genome still accommodating two divergent parental histories [51,52,53].

## 5. Conclusions

This study generated a grass carp × yellowcheek allotriploid and provides a multi-tissue view of parental genome contributions in a newly formed animal polyploid. CCE showed broad maternal-biased expression associated with the expected 2:1 maternal-to-paternal genomic dosage, together with tissue-specific paternal contributions. These findings provide insight into how genomic dosage and tissue-dependent regulation shape early expression divergence after hybridization and triploidization. The optimized hydrostatic pressure protocol also provides a methodological reference for producing sterile polyploid fish materials. Nevertheless, several questions remain to be explored in future studies, such as the developmental dynamics of parental genome regulation and the functional consequences of gut-related expression changes under controlled feeding or physiological conditions.

## Figures and Tables

**Table 1:** Optimization of hydrostatic pressure and shock initiation timing for triploidy induction.

Treatment Duration (2 Min)	Control	34 MPa	48 MPa	68 MPa	Post-Fertilization Time
**Fertilization Rate**	91%	93%	92%	68%	2.5 Min
**Hatching Rate**	73%	62%	71%	3%	
**Triploidy Rate**	0%	80%	100%	100%	
**Fertilization Rate**	94%	97%	96%	80%	3.5 Min
**Hatching Rate**	77%	65%	70%	5%	
**Triploidy Rate**	0%	80%	100%	100%	

Note: MPa, megapascal; min, minute; fertilization rate, percentage of fertilized embryos among examined embryos; hatching rate, percentage of hatched larvae under each treatment condition; triploidy rate, percentage of triploid larvae among examined larvae. Values are descriptive percentages calculated from embryo or larval counts for each treatment condition.

**Table 2:** Optimization of treatment duration for triploidy induction.

Treatment Duration	1.5 Min	2 Min	2.5 Min
**Fertilization Rate**	96%	96%	96%
**Hatching Rate**	72%	70%	66%
**Triploidy Rate**	100%	100%	100%

Note: MPa, megapascal; min, minute; fertilization rate, percentage of fertilized embryos among examined embryos; hatching rate, percentage of hatched larvae under each treatment condition; triploidy rate, percentage of triploid larvae among examined larvae. Values are descriptive percentages calculated from embryo or larval counts for each treatment condition.

## Data Availability

The transcriptome raw data generated in this study have been deposited in the NCBI BioProject database under accession number PRJNA1416878. All other data are available within the article and its Supplementary Materials.

## Supplementary Materials

The following supporting information can be downloaded at https://www.mdpi.com/article/10.3390/ani16152299/s1, Figure S1: Representative metaphase chromosome of the triploid CCE. Scale bar, 10 μm; Figure S2. KEGG enrichment of novel ELD-*Eb* genes in brain and hypothalamus. Bubble size indicates gene count, and color indicates the enrichment *p*-value. Table S1: Primer sequences used in this study. Table S2: Homeolog pair ID, allele-specific expression (ASE) counts, and parental bias [log2(*Eb*/*Ci*)] of 18,094 homeolog pairs across seven tissues in triploid CCE; Table S3: Gene ID, expression pattern (Class), and category (1–12) of 7729 DEGs identified in the gut of triploid CCE; Table S4: Gene ID, expression pattern (Class), and category (1–12) of 10,408 DEGs identified in the brain of triploid CCE; Table S5: Gene ID, expression pattern (Class), and category (1–12) of 8533 DEGs identified in the kidney of triploid CCE; Table S6: Gene ID, expression pattern (Class), and category (1–12) of 6558 DEGs identified in the liver of triploid CCE; Table S7: Gene ID, expression pattern (Class), and category (1–12) of 5454 DEGs identified in the muscle of triploid CCE; Table S8: Gene ID, expression pattern (Class), and category (1–12) of 8785 DEGs identified in the spleen of triploid CCE; Table S9: Gene ID, expression pattern (Class), and category (1–12) of 9862 transcripts identified in the hypothalamus of triploid CCE.

## References

[1] Comai L. (2005). The advantages and disadvantages of being polyploid. Nat. Rev. Genet..

[2] Zhou L., Gui J.F. (2023). Epigenetics in hybridization and polyploidization of aquatic animals. Epigenetics in Aquaculture.

[3] Salman-Minkov A., Sabath N., Mayrose I. (2016). Whole-genome duplication as a key factor in crop domestication. Nat. Plants.

[4] Leitch A.R., Leitch I.J. (2008). Genomic plasticity and the diversity of polyploid plants. Science.

[5] Zhou L., Gui J.F. (2017). Natural and artificial polyploids in aquaculture. Aquac. Fish..

[6] Lu M., Zhang Q.C., Zhu Z.Y., Peng F., Li Z., Wang Y., Li X.Y., Wang Z.W., Zhang X.J., Zhou L. (2023). An efficient approach to synthesize sterile allopolyploids through the combined reproduction mode of ameiotic oogenesis and sperm-egg fusion in the polyploid *Carassius* complex. Sci. Bull..

[7] Ren L., Luo M., Cui J., Gao X., Zhang H., Wu P., Wei Z., Tai Y., Li M., Luo K. (2025). Variation and interaction of distinct subgenomes contribute to growth diversity in intergeneric hybrid fish. Genom. Proteom. Bioinform..

[8] Roche J., Griot R., Allal F., Besson M., Haffray P., Patrice P., Phocas F., Vandeputte M. (2024). APIS: An updated parentage assignment software managing triploids induced from diploid parents. G3.

[9] Aufiero G., Fruggiero C., D'Angelo D., D'Agostino N. (2024). Homoeologs in allopolyploids: Navigating redundancy as both an evolutionary opportunity and a technical challenge—A transcriptomics perspective. Genes.

[10] Dong G., Shen J., Zhang Q., Wang J., Yu Q., Ming R., Wang K., Zhang J. (2018). Development and applications of chromosome-specific cytogenetic BAC-FISH probes in *S. spontaneum*. Front. Plant Sci..

[11] Gui J.F. (2024). Chinese wisdom and modern innovation of aquaculture. Water Biol. Secur..

[12] Piferrer F., Beaumont A., Falguière J.-C., Flajšhans M., Haffray P., Colombo L. (2009). Polyploid fish and shellfish: Production, biology and applications to aquaculture for performance improvement and genetic containment. Aquaculture.

[13] Deb S.K., Edger P.P., Pires J.C., McKain M.R. (2023). Patterns, mechanisms, and consequences of homoeologous exchange in allopolyploid angiosperms: A genomic and epigenomic perspective. New Phytol..

[14] Fox D.T., Soltis D.E., Soltis P.S., Ashman T.L., Van de Peer Y. (2020). Polyploidy: A biological force from cells to ecosystems. Trends Cell Biol..

[15] Jackson S.A. (2017). Epigenomics: Dissecting hybridization and polyploidization. Genome Biol..

[16] Chen Z.J. (2007). Genetic and epigenetic mechanisms for gene expression and phenotypic variation in plant polyploids. Annu. Rev. Plant Biol..

[17] Dong Y., Hu G., Zhu T., Liu C., Wendel J.F., Chen Z.J. (2022). Parental legacy versus regulatory innovation in salt stress responsiveness of allopolyploid cotton (*Gossypium*) species. Plant J..

[18] Yoo M., Liu X., Pires J.C., Soltis P.S., Soltis D.E. (2014). Nonadditive gene expression in polyploids. Annu. Rev. Genet..

[19] Session A.M., Uno Y., Kwon T., Chapman J.A., Toyoda A., Takahashi S., Rokhsar D.S. (2016). Genome evolution in the allotetraploid frog *Xenopus laevis*. Nature.

[20] Li J.T., Wang Q., Huang Yang M.D., Li Q.S., Cui M.S., Dong Z.J., Wang X.Y. (2021). Parallel subgenome structure and divergent expression evolution of allo-tetraploid common carp and goldfish. Nat. Genet..

[21] Soltis D.E., Visger C.J., Marchant D.B., Soltis P.S. (2016). Polyploidy: Pitfalls and paths to a paradigm. Am. J. Bot..

[22] Wang Y., Liu W., Li Z., Qiu B., Li J., Geng G., Hu B., Liao A., Cai Y., Wen M. (2024). Improvement and application of genetic resources of grass carp (*Ctenopharyngodon idella*). Reprod. Breed..

[23] Šetlíkova I. (2006). A review of grass carp use for aquatic weed control and its impact on water bodies. J. Aquat. Plant Manag..

[24] Liu X.G., Shen H.Y., Gu Z.J., Cheng G., Wang J., Zhu H. (2023). The environmental impact and development direction of grass carp, *Ctenopharyngodon idella*, aquaculture. J. World Aquac. Soc..

[25] E Z., Wen H., Tang Y., Zhang M., Wang Y., Liao S., Chen K., Lu D., Lin H., Huang W. (2025). Induction of triploid grass carp (*Ctenopharyngodon idella*) and changes in embryonic transcriptome. Animals.

[26] He W., Xie L., Li T., Liu S., Xiao J., Hu J., Wang J., Qin Q., Liu Y. (2013). The formation of diploid and triploid hybrids of female grass carp × male blunt snout bream and their 5S rDNA analysis. BMC Genet..

[27] Li S., Xiong X., Qiu S., Shen Z., He Y., Gao Z., Wan S. (2024). Chromosome-level genome assembly of the yellow-cheek carp Elopichthys bambusa. Sci. Data.

[28] Cassani J.R., Caton W.E. (1986). Efficient production of triploid grass carp (*Ctenopharyngodon idella*) utilizing hydrostatic pressure. Aquaculture.

[29] Yang X.L., Wang Y., Li Z., Lin Q.H., Yu P., Lu M., Li X.Y., Wang Z.W., Zhang X.J., Gui J.F. (2025). Expression pattern changes of three homeologs in chemokine activity enhance antiviral response to herpesvirus infection in a newly synthesized alloheptaploid. BMC Genom..

[30] Li Z., Liang H.W., Wang Z.W., Zou G.W., Gui J.F. (2016). A novel allotetraploid gibel carp strain with maternal body type and growth superiority. Aquaculture.

[31] Zhu H.P., Gui J.F. (2007). Identification of genome organization in the unusual allotetraploid form of *Carassius auratus* gibelio. Aquaculture.

[32] West A.C., Wood S.H., Iversen M., van Dalum M.J., Jørgensen E.H., Sandve S.R., Hazlerigg D.G. (2026). Photoperiodic history modulates the response of the saccus vasculosus transcriptome to seawater exposure in Atlantic salmon. J. Comp. Physiol. A.

[33] Wu C.S., Ma Z.Y., Zheng G.D., Zou S.-M., Zhang X.J., Zhang Y.A. (2022). Chromosome-level genome assembly of grass carp (*Ctenopharyngodon idella*) provides insights into its genome evolution. BMC Genom..

[34] Li H., Handsaker B., Wysoker A., Fennell T., Ruan J., Homer N., Marth G., Abecasis G., Durbin R. (2009). The sequence Alignment/Map format and SAMtools. Bioinformatics.

[35] Rapp R.A., Udall J.A., Wendel J.F. (2009). Genomic expression dominance in allopolyploids. BMC Biol..

[36] Almeida-Silva F., Prost-Boxoen L., Van de Peer Y. (2024). Hybridexpress: An R/Bioconductor package for comparative transcriptomic analyses of hybrids and their progenitors. New Phytol..

[37] McKenna A., Hanna M., Banks E., Sivachenko A., Cibulskis K., Kernytsky A., Garimella K., Altshuler D., Gabriel S., Daly M. (2010). The genome analysis toolkit: A mapreduce framework for analyzing next-generation DNA sequencing data. Genome Res..

[38] Wittkopp P.J., Haerum B.K., Clark A.G. (2004). Evolutionary changes in cis and trans gene regulation. Nature.

[39] Wang Y., Lu Y., Zhang Y., Ning Z., Li Y., Zhao Q., Lu H., Huang R., Xia X., Feng Q. (2015). The draft genome of the grass carp (*Ctenopharyngodon idellus*) provides insights into its evolution and vegetarian adaptation. Nat. Genet..

[40] McCarter N.H. (1988). Verification of the production of triploid grass carp (*Ctenopharyngodon idella*) with hydrostatic pressure. N. Z. J. Mar. Freshw. Res..

[41] Li W., Liu J., Tan H., Luo L., Cui J., Hu J., Wang S., Liu Q., Hu F., Tang C. (2018). Asymmetric expression patterns reveal a strong maternal effect and dosage compensation in polyploid hybrid fish. BMC Genom..

[42] Ren L., Tang C., Li W., Cui J., Tan X., Xiong Y., Chen J., Wang J., Xiao J., Zhou Y. (2017). Determination of dosage compensation and comparison of gene expression in a triploid hybrid fish. BMC Genom..

[43] Ren L., Yan X., Cao L., Li J., Zhang X., Gao X., Liu J., Cui J., Liu S. (2019). Combined effects of dosage compensation and incomplete dominance on gene expression in triploid cyprinids. DNA Res..

[44] Matos I., Machado M.P., Schartl M., Coelho M.M. (2015). Gene expression dosage regulation in an allopolyploid fish. PLoS ONE.

[45] Birchler J.A., Veitia R.A. (2012). Gene balance hypothesis: Connecting issues of dosage sensitivity across biological disciplines. Proc. Natl. Acad. Sci. USA.

[46] Nieto Feliner G., Casacuberta J., Wendel J.F. (2020). Genomics of evolutionary novelty in hybrids and polyploids. Front. Genet..

[47] Soltis P.S., Marchant D.B., Van de Peer Y., Soltis D.E. (2015). Polyploidy and genome evolution in plants. Curr. Opin. Genet. Dev..

[48] Limwachirakhom R., Triwutanon S., Chumkam S., Jintasataporn O. (2022). Effects of chromium-L-methionine in combination with a zinc amino acid complex or selenomethionine on growth performance, intestinal morphology, and antioxidative enzymes in red tilapia *Oreochromis* spp. Animals.

[49] Salinas I. (2015). The mucosal immune system of teleost fish. Biology.

[50] Zhou C., Wang Z., Ran M., Liu Y., Song Z. (2024). Nano-selenium ameliorates microplastics-induced injury: Histology, antioxidant capacity, immunity and intestinal microbiota of grass carp (*Ctenopharyngodon idella*). Ecotoxicol. Environ. Saf..

[51] Chen Z.J. (2010). Molecular mechanisms of polyploidy and hybrid vigor. Trends Plant Sci..

[52] Chen Z.J. (2013). Genomic and epigenetic insights into the molecular bases of heterosis. Nat. Rev. Genet..

[53] Mable B.K. (2013). Polyploids and hybrids in changing environments: Winners or losers in the struggle for adaptation?. Heredity.

