# Supplementary material for: Dosage-Associated Maternal Dominance and Tissue-Specific Paternal Expression in a Triploid Hybrid Between Grass Carp and Yellowcheek
Source: Animals (Basel). 2026 Jul 24;16(15):2299. doi: 10.3390/ani16152299 (PMC13463558; doi:10.3390/ani16152299)

Figure S2. KEGG enrichment of novel ELD-Eb genes in brain and hypothalamus. Bubble size indicates gene count, and color indicates the enrichment P value.

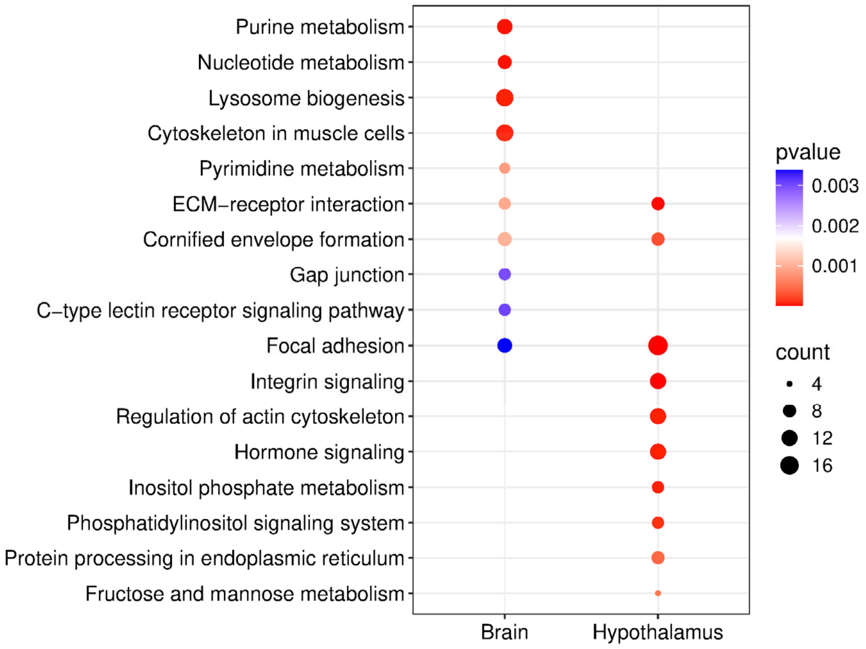

Supplement: Supplementary file 1 [file animals-16-02299-s001.zip › Figure S2.pdf]
